# Immediate reduction under general anesthesia and combined anterior and posterior fusion in the treatment of distraction-flexion injury in the lower cervical spine

**DOI:** 10.1186/s13018-018-0842-x

**Published:** 2018-05-29

**Authors:** De-chao Miao, Feng Wang, Yong Shen

**Affiliations:** Department of Spine Surgery, The Third Hospital of Hebei Medical University, 139 Ziqiang Road, Shijiazhuang, 050051 China

**Keywords:** Lower cervical spine, Spinal cord injury, Distraction-flexion, Immediate reduction, Anterior cervical approach, Posterior cervical approach

## Abstract

**Background:**

Distraction-flexion of the lower cervical spine is a severe traumatic lesion, frequently resulting in paralysis. The optimal surgical treatment is controversial. It has been a challenge for orthopedic surgeons to manage distraction-flexion injury in the lower cervical spine while avoiding the risk of iatrogenic damage. Thus, safer strategies need to be designed and adopted.This study aimed to evaluate the clinical efficacy of immediate reduction under general anesthesia and combined anterior and posterior fusion in the treatment of distraction-flexion injury in the lower cervical spine.

**Methods:**

Twenty-four subjects of traumatic lower cervical spinal distraction-flexion were retrospectively analyzed from January 2010 to December 2013. Traffic accident was the primary cause of injury, with patients presenting with dislocated segments in C4–5 (*n* = 8), C5–6 (*n* = 10), and C6–7 (*n* = 6). Sixteen patients had unilateral facet dislocation and eight had bilateral facet dislocation. Spinal injuries were classified according to the American Spinal Injury Association (ASIA) impairment scale (2000 edition amended), with four cases of grade A, four cases of grade B, ten cases of grade C, four cases of grade D, and two cases of grade E. On admission, all patients underwent immediate reduction under general anesthesia and combined anterior and posterior fusion. The mean follow-up time was 3.5 years.

**Results:**

All operations were completed successfully, with no major complications. Postoperative X-rays showed satisfactory height for the cervical intervertebral space and recovery of the vertebral sequence. Bone fusion was completed within 4 to 6 months after surgery. Surgery also significantly improved neurological function in all patients.

**Conclusion:**

Immediate reduction under general anesthesia and combined anterior and posterior fusion can be used to successfully treat distraction-flexion injury in the lower cervical spine, obtaining completed decompression, safe spinal re-alignment, and excellent immediate postoperative stability.

## Background

Lower cervical injury is the most common type of all injuries to the cervical spine, with lower cervical facet dislocation accounting for 6 to 15% [1]. This type of injury mainly involves excessive flexion-distraction or flexion-rotation, present as the subluxation or dislocation of the facet joints and may be accompanied by spinal cord injuries [2]. Distraction-flexion of the lower cervical spine is most commonly caused by road traffic accidents and most frequently affects the levels C5–6 and C6–7 [3]. The injuries can result in significant impact on neurological function, high economic cost, and may at times be life-threatening.

The goal of treatment is to restore the normal architecture of the cervical spine, recover the anatomical and functional integrity of the spinal cord and nerve root, completely decompress and restore the intervertebral height and physiological curvature, and avoid delayed or secondary neurological injury for immediate and long-term stability of the cervical spine [4–7]. Methods described to treat distraction-flexion patients include closed traction, Halo thoracic brace, anterior or posterior approach, or both [3, 8]. However, to date, the treatment has not been standardized. The aim of the current study was to examine the clinical efficacy of immediate reduction under general anesthesia and combined anterior and posterior fusion in the treatment of distraction-flexion injury in the lower cervical spine to provide evidence for clinical strategies.

## Methods

### Patients

We retrospectively reviewed our experience using immediate reduction under general anesthesia followed by antero-posterior fixation in the treatment of distraction-flexion injury in the lower cervical spine during a consecutive 4-year period (from January 2010 to December 2013). The inclusion criteria consisted of unilateral or bilateral facet dislocations, with disc herniation existed both anteriorly and posteriorly, or unstable 3-column injuries of lower cervical spine. We enrolled a final cohort of 24 patients (14 males, 10 females), who were diagnosed with distraction-flexion injury in the lower cervical spine. Patients’ age ranged from 21 to 68 years, with a mean age of 44.42 years. The etiology of trauma included traffic accidents (18 patients), high falls (2 patients), and others (2 patients). All patients were imaged using cervical X-rays, CT scanning, and MRI of the cervical spine. Plain radiography and CT showed facet dislocations at C4–5 (8 patients), C5–6 (10 patients), and C6–7 (6 patients). Sixteen cases presented with unilateral facet dislocation and eight cases with bilateral facet dislocation. Two patients presented with intact neurological function, 4 patients with complete spinal cord injury, and 18 patients with incomplete spinal cord injury. As per the classifications of the American Spinal Injury Association (ASIA) impairment scale [9], encompassing complete injury (grade A) to normal (grade E), we found that four cases were grade A, four cases were grade B, ten cases were grade C, four cases were grade D, and two cases were grade E (Table 1). Patients with clinical evidence of spinal cord injury accepted a methylprednisolone sodium succinate according to the National Acute Spinal Cord Injury StudyII protocol [2]. All patients were operated within 72 h following the injury. All surgeries ranged from 4 to 7 h in duration.

**Table 1:** General data of enrolled cases

Case no.	Age (year)	Sex (male/female)	Involved segment	Unilateral/bilateral	Spinal cord injury	Time to surgery (h)	Traction weight (kg)	Time of reduction (min)
1	33	M	C6–7	U	Incomplete spinal cord injury	12	11	60
2	49	F	C4–5	U	Incomplete spinal cord injury	14	10	50
3	68	M	C6–7	B	Complete spinal cord injury	72	12	70
4	21	F	C4–5	U	Intact neurological function	8	9	40
5	45	M	C4–5	B	Incomplete spinal cord injury	52	10	50
6	58	F	C5–6	U	Incomplete spinal cord injury	32	10	50
7	54	M	C5–6	B	Complete spinal cord injury	26	11	60
8	46	F	C4–5	U	Incomplete spinal cord injury	39	9	40
9	37	M	C6–7	U	Incomplete spinal cord injury	40	12	70
10	38	F	C4–5	B	Incomplete spinal cord injury	44	9	40
11	50	M	C5–6	U	Incomplete spinal cord injury	30	11	60
12	34	F	C4–5	U	Incomplete spinal cord injury	32	10	50
13	29	M	C5–6	U	Incomplete spinal cord injury	16	12	70
14	50	M	C6–7	B	Complete spinal cord injury	48	11	60
15	42	M	C5–6	U	Incomplete spinal cord injury	36	9	40
16	39	F	C4–5	U	Incomplete spinal cord injury	23	10	50
17	56	F	C5–6	B	Complete spinal cord injury	30	9	40
18	28	M	C5–6	U	Intact neurological function	24	11	60
19	44	M	C4–5	U	Incomplete spinal cord injury	34	10	70
20	64	F	C5–6	B	Incomplete spinal cord injury	64	12	70
21	55	M	C6–7	U	Incomplete spinal cord injury	48	11	60
22	42	M	C5–6	U	Incomplete spinal cord injury	28	12	70
23	48	F	C5–6	B	Incomplete spinal cord injury	28	11	60
24	36	M	C6–7	U	Incomplete spinal cord injury	40	11	60
Average	44.42					34.17	10.54	56.25

### Surgical technique

A neck collar was applied to patients in the supine position. Following general anesthesia, the neck collar was removed and patients were moved into a position of mild cervical flexion. Spinal cord evoked potential monitoring was introduced to monitor the patient’s neurological function during reduction. Intraoperative X-ray fluoroscopy was used to observe the reduction process. Skull traction was performed with 5 kg weights, and the weight was increased at a rate of 1 kg per 10 min. During each interval, patients’ limb neurological function changes were closely monitored and the reset situation was observed through intraoperative X-ray fluoroscopy. When the upper and lower articular process was pulled to the apex of the tip to the tip, the unilateral facet dislocation was unlocked by slight stretching of the head, allowing the inferior articular process of the dislocated vertebra to cross the superior process of the lower vertebra. Slight rotation of the neck toward the dislocated side allowed the bilateral facet dislocation to be reset. The traction weight was then gradually reduced to 5 kg after reduction. Traction was stopped in cases where the traction weight exceeded 15 kg, the dislocation was unable to be reset, or neurological deterioration was observed through spinal cord evoked potential monitoring.

A standard Smith-Robinson anterior approach [10] was used to perform anterior decompression and fixation after closed traction. Plate with screw fixation and inter-body cages were used for fusion. For patients who failed closed reduction, a Caspar distractor was used to distract the intervertebral space after discectomy followed by a thin distractor for poking to achieve reduction. Then, the patient was turned to the prone position, the dislocated spinous process was fastened by lateral mass or pedicle screws and rods, fusion consisted of excising the articular cartilage and filling the articular gap with autogenous or allograft bone. Posterior decompression was also done if necessary.

**Fig. 1:**
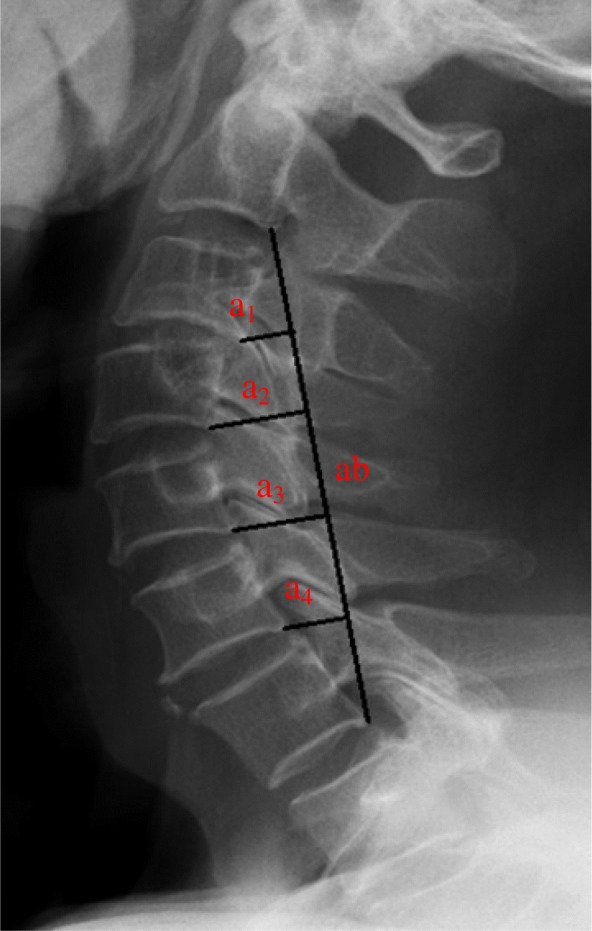
Calculation of CCI. “ab” was the line connecting posterior inferior edge of the C2 and C7 vertebral body. “a1” to “a4” respectively represented the vertical distance from posterior inferior edge of the C3-C6 to “ab.” CCI = [(a1 + a2 + a3 + a4)/ab] × 100%

A neck collar was used for 1 to 1.5 months postoperatively. All patients were graded before and after surgery according to the Japanese Orthopedic Association (JOA) score and ASIA grades to evaluate the neurological state, and the cervical curvature index (CCI) (Fig. 1) was measured pre-and postoperatively to evaluate the stability of the cervical spine.

### Statistical method

SPSS 22.0 statistical software (IBM, Armonk, NY, USA) was used for statistical analysis. Data was recorded as mean ± SD and was compared by using a *t* test. Wilcoxon rank test was applied to analyze ASIA grades that recorded preoperatively and at the latest follow-up visit. *P* value < 0.05 was considered statistically significant.

## Results

The skull traction weight ranged from 9 to 12 kg, with a mean of 10.54 kg. Reduction was achieved after 40 to 70 min of traction (mean, 56.25 min). All subjects got reduction except two patients who got reduction incompletely.

The surgery length was 328.33 ± 47.88 min, and the amount of blood loss was 734.58 ± 96.68 ml. All patients were followed up for 3 to 6 years. Bone fusion was completed within 4 to 6 months after surgery (obvious fiber through and bone connections can be seen in the X-ray and CT scans).

No severe complications were noted for any of the patients. None of the patients showed plate fracture, screw loosening, cage prolapse, or pseudarthrosis at the follow-up. X-ray examination showed satisfactory recovery of the cervical intervertebral height space and vertebral sequence.

**Fig. 2:**
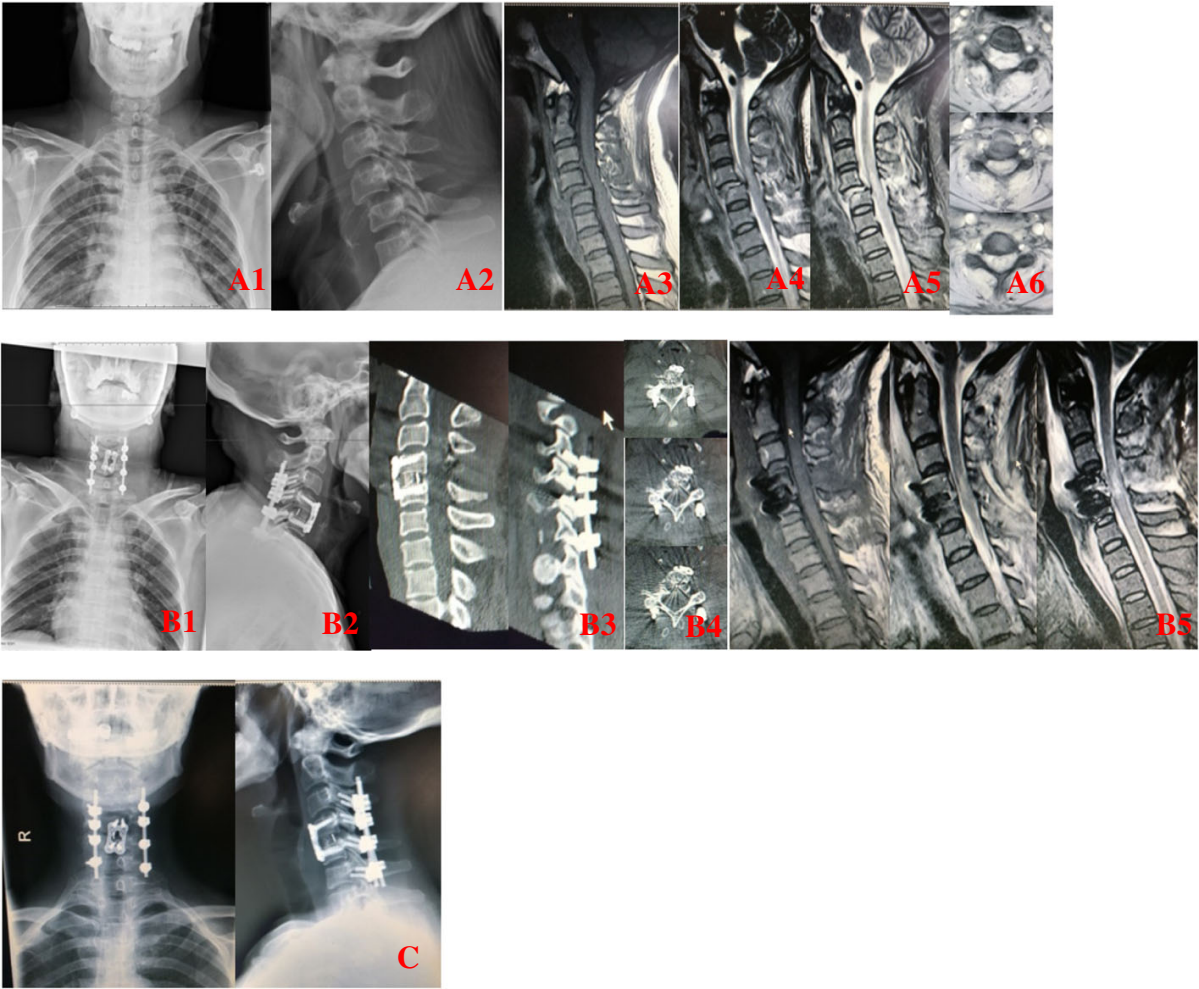
Pre- and postoperative JOA grade and cervical curvature index (CCI) and ASIA grade

Neurological function was also significantly improved at the follow-up as compared with preoperative values (Table 2). The paralysis plane for patients with complete spinal cord injury did not increase after surgery. Neurological function in patients with incomplete spinal cord injury was restored to varying degrees. The postoperative JOA scores and CCI showed improvement as compared with preoperative (Table 3, Fig. 2).

**Table 2:** Pre- and postoperative ASIA grade

ASIA grade	Pre-op cases	The last follow-up ASIA grade				
		A	B	C	D	E
A	4	1	2	1		
B	4		1	1	2	
C	10			2	4	4
D	4				1	3
E	2					2

**Table 3:** Pre- and postoperative JOA grade and cervical curvature index (CCI) and ASIA grade

	Preoperative	The last follow-up	*p*	Improvement rate of JOA grade (%)
JOA grade	9.21 ± 4.38	13.17 ± 4.01	0.000	54.88 ± 33.72
CCI	18.90 ± 0.91	10.60 ± 0.43	0.000	
ASIA grade			0.010

**Fig. 3:**
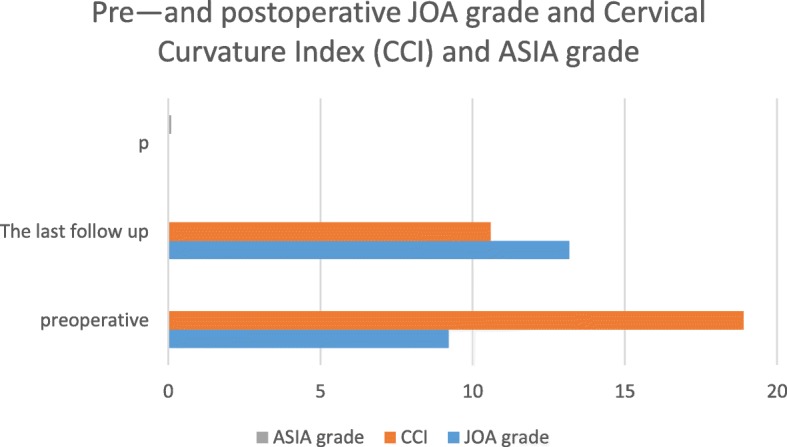
A typical case imaging data. A1-A6, lateral view of radiographs demonstrated bilateral facet dislocation of C4-C5. MRI images showing disc herniation existed both anteriorly and posteriorly. B1–5, stabilization was performed via an anterior-posterior cervical approach with discectomy and fusion with inter-body cage, allograft, and Synthes plate; CT and MRI show good alignment and satisfactory decompression. C, last follow-up X-ray shows good alignment and union of allograft with the adjacent vertebral bodies

Although some patients complained of slight neck stiffness and discomfort postoperatively, none of them complained of neckache, limited neck activity, and a sore back. Figure 3 shows typical case imaging data.

## Discussion

Distraction-flexion injuries of the lower cervical spine are usually accompanied by a disruption to the anterior or posterior elements, such as the longitudinal ligaments, the ligamentum flavum, apophyseal joint ligaments, the annulus fibrosus, and the interspinous ligaments [11], which could cause instability of the lower cervical spine. The goals of treatment of the lower cervical spine injury include a return to the normal architecture of the cervical spine, a minimum of residual pain, a recover of the functional integrity of the spinal cord, and the prevention of delayed or secondary disability [12]. However, to date, the treatment has not been standardized, and there remain several unanswered questions with regard to treatment [13]. We performed an immediate reduction under general anesthesia and followed by a combined anterior and posterior fusion and fixation in 24 patients with distraction-flexion injuries of the lower cervical spine. Bone fusion and postoperative re-alignment were obtained in all patients and maintained throughout the follow-up period.

Manually closed reduction is usually initially adopted, which is a basis on which the next steps are based [13]. On the other hand, it is comprehensively accepted that cervical spine dislocations should be reduced as early as possible by closed means, because this has great impact on neurologic recovery [14]. The most common form of initial reduction has been always an attempt at awake closed reduction with skull tongs. However, awake closed reduction has some drawbacks, such as requires heavy traction weights, exposes the patients to unbearable immobilization and pain, or may cause secondary neurological injury [15–17]. However, some biomechanical studies have demonstrated the safety of skull traction [18–20]. In our study, we performed immediate reduction under general anesthesia with spinal cord evoked potential monitoring, which ensured the safety of the closed traction procedure. During the process of traction, when the upper and lower articular process was pulled to the apex of the tip to the tip, the unilateral facet dislocation was unlocked by slight stretching of the head, allowing the inferior articular process of the dislocated vertebra to cross the superior process of the lower vertebra while slight rotation of the neck toward the dislocated side allowed the bilateral facet dislocation to be reset. In this method, all subjects got reduction (two patients got reduction incompletely) and making it possible to manage the combined anterior and posterior fusion and fixation after traction.

Open reduction can be achieved through an anterior approach alone, a posterior approach alone, or a combined anterior and posterior approach [21]: the surgical approach is not standardized. Previously, anterior reduction alone was commonly used for facet dislocation patients, because anterior approach rarely causes iatrogenic soft tissue injury as it reaches the injury more directly. In addition, decompression can be achieved with direct observation of anterior pathology including rupture of the anterior longitudinal ligament or herniation or rupture of the nucleus pulposus [22]. Maynard et al. reported a series of direct anterior open reduction of distraction-flexion injuries without attempting closed reduction [23]; their procedure is very similar to the one used in our study. The increased use of an anterior approach has meant that its indication is no longer limited to injuries of anterior structures, with posterior injuries also treatable. However, Henriques et al*.* reported that anterior fixation alone has a lower fusion rate; 7/13 patients achieve inadequate fusion for bilateral dislocation [24]. However, many other scholars also suggest that anterior fixation alone is less effective in bilateral dislocations or cervical spine injuries with severe instability than combined approach [22, 24–26]. And a potential problem after anterior approach alone is postoperative kyphosis. Concern about the mechanical failure of distraction-flexion injuries should be high; hence, a combined anterior and posterior fusion and fixation was recommended for patients with distraction-flexion injuries, especially for patients with fractures of both facets and endplate.

Posterior open reduction can be obtained by distracting the two dislocated spinous processes with two bone-holding forceps and, if necessary, simultaneously poking the facets with a narrow osteotome. When satisfactory reduction was achieved, the cervical spine was moved into slight extension, and the dislocated spinous process fastened by lateral mass or pedicle screws. This method is a good alternative for treating distraction-flexion injuries; additionally, patients with posterior fracture or compression associated with facet dislocation, such as a lamina fragment into the canal, can be successfully treated by using a posterior approach [27]. However, posterior approach alone may add some risk of neurological deterioration in patients with anterior compression and less likely to restore cervical lordosis. Therefore, one-stage operation in combination with an anterior and posterior fusion and fixation was adopted in our study, and all patients showed evidence of stability and neurological recovery on the final follow-up examination, which is consistent with the conclusions of many studies [28–30].

The combined approach can provide the strongest internal fixation and significantly limiting motion [27]. A combined anterior and posterior fixation of the lower cervical dislocation increase the bone fusion rate, and it is helpful to restore cervical alignment and neural decompression, especially for patients with chronic injuries associated with pseudoarthrosis. A combined approach is also recommended to patients with poor bone quality, such as those with ankylosing spondylitis, osteoporosis, or other chronic conditions. However, benefits of a combined approach must be weighed against the risk of an addition surgery and increasing morbidity related to each approach as well as increasing surgical costs [29].

The timing of the surgery is another controversial aspect of this treatment. Cervical facet dislocation should be reduced as soon as possible, as recommended in the guidelines published in 2013 [31]. However, there are some risks associated with early reduction, such as neurological deterioration caused by a herniated disc or vertebral artery injury with posterior circulation stroke [32, 33]. Nagata et al. indicated that early reduction of cervical spine dislocation (≤ 6 h of injury) might facilitate motor function improvement, even in patients with complete motor paralysis [34]. In contrast, Vaccaro suggested that a delay in surgery allows for better preoperative preparation and that decompression is safer after the edema has subsided [35]. Most authors accept that rapid reduction should give patients with distraction-flexion injuries the chance for neurological recovery or at least prevent progressive secondary spinal cord injury if the patient’s condition allows. We recommend that surgery should be performed within 72 h of injury if the patient’s condition permits, which is conducive to the recovery of neurological function.

The recovery of neurological injury was examined by comparing the difference in pre-operative ASIA grade and JOA scores of motor and sensory function in our study. No neurological worsening occurred; neurological function in patients with incomplete spinal cord injury was restored to varying degrees, yet symptoms of nerve root irritation had disappeared. Considering our result and previous report, an effective reduction decompression and internal fixation system for lower cervical distraction-flexion relieve neurological deterioration, provide immediate stabilization, enhance bony fusion, and correct the spine deformity [36]. However, the choice of surgical approach in the treatment of traumatic cervical dislocation is highly variable and may be influenced by a variety of factors. Nassr et al. conducted a retrospective survey analysis of surgical approach in treatment of lower cervical distraction-flexion and found that combined approach is recommended for the treatment of bilateral dislocation [29], which is in line with our findings.

In this study, we measured the CCI as described by Ishihara [37] (Fig. 1) to evaluate the stability of the cervical spine. The biomechanical superiority of posterior instrumentation and a high stability of cervical lateral mass or pedicle screws in cervical trauma have been reported [38]. However, Brodke et al. found no significant difference in stability between patients treated via an anterior approach [3]. On the other hand, Du et al. suggested that reconstruction of cervical lordosis and strengthening of cervical stability can reduce the incidence of axial symptoms [39]. As such, CCI is an important measure for evaluating the efficacy of the postoperative effect of patients with cervical spinal cord injury. We think that the CCI can also be used to predict the occurrence of adjacent segment disease after cervical surgery. More relevant studies are needed to prove its clinical significance.

The limitation of the current study is the small number of cases. However, we gained precious experience from the use of combined anterior and posterior fusion and fixation, which enable us to continuously improve and consummate the treatment of distraction-flexion injuries of the lower cervical spine.

## Conclusion

Immediate reduction under general anesthesia and combined anterior and posterior fusion can be used to successfully treat distraction-flexion injury in the lower cervical spine. Complete decompression, good reduction, and recovery of the intervertebral height and curvature of the spine can be achieved through this method.

## Abbreviations

ASIA: American Spinal Injury Association
CCI: Cervical curvature index
JOA: Japanese Orthopedic Association
